# Supplementary material for: A proof-of-concept point-of-care test for the serodiagnosis of human amebic liver abscess
Source: PeerJ. 2025 Mar 31;13:e19181. doi: 10.7717/peerj.19181 (PMC11967440; doi:10.7717/peerj.19181)
Supplement: Supplemental Information 1 — Direct comparison results between ELISA OD ranges and ICT grades. [file peerj-13-19181-s001.pdf]

Table S1. Results of the detection by the Amebiasis-ICT and ELISA. (N = 253)

No.	Sample Name	Amebiasis detection	
		**Amebiasis-ICT** (color intensity of T-line)	**ELISA method using the EhHK9** antigen (OD_{490})
1	Amebic liver abscess 1	4	1.381
2	Amebic liver abscess 2	3	1.641
3	Amebic liver abscess 3	3	1.304
4	Amebic liver abscess 4	3	0.870
5	Amebic liver abscess 5	4	1.240
6	Amebic liver abscess 6	3	1.594
7	Amebic liver abscess 7	4	1.487
8	Amebic liver abscess 8	3	0.921
9	Amebic liver abscess 9	3	1.385
10	Amebic liver abscess 10	1	0.433
11	Amebic liver abscess 11	4	1.899
12	Amebic liver abscess 12	1	0.734
13	Amebic liver abscess 13	2	0.726
14	Toxocariasis 1	0	0.173
15	Schistosomiasis mekongi 1	0	0.351
16	Schistosomiasis mekongi 2	3	0.450
17	Schistosomiasis mekongi 3	0	0.148
18	Schistosomiasis mekongi 4	0	0.210
19	Schistosomiasis mekongi 5	0	0.265
20	Schistosomiasis mekongi 6	1	0.333
21	Schistosomiasis mekongi 7	0	0.140
22	Schistosomiasis mekongi 8	0	0.242
23	Schistosomiasis mekongi 9	0	0.155
24	Schistosomiasis mekongi 10	0.5	0.173
25	Clonorchisais 1	N	0.111
26	Clonorchisais 2	N	0.182
27	Clonorchisais 3	N	0.240
28	Clonorchisais 4	N	0.079
29	Clonorchisais 5	N	0.177
30	Clonorchisais 6	N	0.270
31	Clonorchisais 7	N	0.231
32	Clonorchisais 8	N	0.205
33	Clonorchisais 9	1	0.488
34	Clonorchisais 10	0.5	0.314
35	Ascariasis 1	0	0.111
36	Ascariasis 2	0	0.086
37	Ascariasis 3	0	0.133
38	Ascariasis 4	0	0.075
39	Ascariasis 5	0	0.157
40	Ascariasis 6	0	0.125
41	Ascariasis 7	0	0.150
42	Ascariasis 8	0	0.064
43	Ascariasis 9	0	0.106
44	Ascariasis 10	0	0.109

45	Hookworm infection 1	0	0.244
46	Hookworm infection 2	0	0.271
47	Hookworm infection 3	0	0.143
48	Hookworm infection 4	1	0.449
49	Hookworm infection 5	0	0.231
50	Hookworm infection 6	0	0.133
51	Hookworm infection 7	0	0.135
52	Hookworm infection 8	0	0.116
53	Hookworm infection 9	0	0.114
54	Hookworm infection 10	0	0.097
55	Trichuriasis 1	0	0.128
56	Trichuriasis 2	0	0.035
57	Trichuriasis 3	0	0.034
58	Trichuriasis 4	0	0.133
59	Trichuriasis 5	0	0.089
60	Trichuriasis 6	0	0.172
61	Trichuriasis 7	0	0.205
62	Trichuriasis 8	0	0.255
63	Trichuriasis 9	0	0.186
64	Trichuriasis 10	0	0.110
65	Capillariasis 1	0	0.139
66	Capillariasis 2	0	0.235
67	Capillariasis 3	0	0.244
68	Capillariasis 4	0	0.156
69	Capillariasis 5	0	0.053
70	Capillariasis 6	0.5	0.524
71	Capillariasis 7	0	0.158
72	Capillariasis 8	0	0.107
73	Capillariasis 9	0	0.150
74	Capillariasis 10	0	0.206
75	Strongyloidiasis 1	0	0.120
76	Strongyloidiasis 2	0	0.088
77	Strongyloidiasis 3	0	0.102
78	Strongyloidiasis 4	0	0.225
79	Strongyloidiasis 5	0	0.150
80	Strongyloidiasis 6	0	0.270
81	Strongyloidiasis 7	0	0.038
82	Strongyloidiasis 8	0	0.247
83	Strongyloidiasis 9	0	0.072
84	Strongyloidiasis 10	0	0.162
85	Opisthorchiasis viverrini 1	0	0.210
86	Opisthorchiasis viverrini 2	0	0.038
87	Opisthorchiasis viverrini 3	0	0.105
88	Opisthorchiasis viverrini 4	0	0.130
89	Opisthorchiasis viverrini 5	0	0.147
90	Opisthorchiasis viverrini 6	0	0.100
91	Opisthorchiasis viverrini 7	0	0.066
92	Opisthorchiasis viverrini 8	0	0.171
93	Opisthorchiasis viverrini 9	0	0.255

94	Opisthorchiasis viverrini 10	0	0.122
95	Fascioliasis 1	1	0.202
96	Fascioliasis 2	0	0.601
97	Fascioliasis 3	0	0.234
98	Fascioliasis 4	0	0.100
99	Fascioliasis 5	0	0.126
100	Fascioliasis 6	0	0.255
101	Fascioliasis 7	0	0.162
102	Fascioliasis 8	0	0.209
103	Fascioliasis 9	0	0.516
104	Fascioliasis 10	0	0.221
105	Angiostrongyliasis 1	0	0.174
106	Angiostrongyliasis 2	0	0.176
107	Angiostrongyliasis 3	0	0.133
108	Angiostrongyliasis 4	0	0.164
109	Angiostrongyliasis 5	0	0.129
110	Angiostrongyliasis 6	0	0.163
111	Angiostrongyliasis 7	0	0.188
112	Angiostrongyliasis 8	0	0.058
113	Angiostrongyliasis 9	0	0.160
114	Angiostrongyliasis 10	0	0.058
115	Gnathostomiasis 1	0	0.294
116	Gnathostomiasis 2	0	0.168
117	Gnathostomiasis 3	0	0.073
118	Gnathostomiasis 4	0	0.127
119	Gnathostomiasis 5	0	0.234
120	Gnathostomiasis 6	0	0.107
121	Gnathostomiasis 7	0	0.179
122	Gnathostomiasis 8	0	0.118
123	Gnathostomiasis 9	0	0.137
124	Gnathostomiasis 10	0	0.110
125	Trichinellosis 1	0	0.093
126	Trichinellosis 2	0	0.115
127	Trichinellosis 3	0	0.172
128	Trichinellosis 4	0	0.177
129	Trichinellosis 5	0	0.125
130	Trichinellosis 6	0	0.100
131	Trichinellosis 7	0	0.148
132	Trichinellosis 8	0	0.135
133	Trichinellosis 9	0	0.182
134	Trichinellosis 10	0	0.065
135	Cysticercosis 1	0	0.136
136	Cysticercosis 2	0	0.412
137	Cysticercosis 3	0	0.279
138	Cysticercosis 4	0	0.109
139	Cysticercosis 5	0	0.027
140	Cysticercosis 6	1	0.247
141	Cysticercosis 7	0	0.174
142	Cysticercosis 8	0	0.075

143	Cysticercosis 9	0	0.202
144	Cysticercosis 10	0	0.068
145	Blastocystosis 1	0	0.318
146	Blastocystosis 2	0	0.009
147	Blastocystosis 3	0	0.154
148	Blastocystosis 4	0	0.163
149	Blastocystosis 5	0	0.124
150	Blastocystosis 6	0	0.177
151	Blastocystosis 7	0	0.157
152	Blastocystosis 8	0	0.102
153	Blastocystosis 9	0	0.120
154	Blastocystosis 10	0	0.098
155	Blastocystosis 11	0	0.124
156	Blastocystosis 12	0	0.724
157	Blastocystosis 13	0	0.082
158	Blastocystosis 14	0	0.280
159	Blastocystosis 15	0	0.116
160	Giardiasis 1	0	0.115
161	Giardiasis 2	0	0.138
162	Giardiasis 3	0	0.250
163	Giardiasis 4	0	0.205
164	Giardiasis 5	0	0.086
165	Giardiasis 6	0	0.212
166	Giardiasis 7	0	0.078
167	Giardiasis 8	0	0.101
168	Giardiasis 9	1	0.263
169	Giardiasis 10	0	0.116
170	Giardiasis 11	0	0.194
171	Giardiasis 12	0	0.188
172	Giardiasis 13	0	0.072
173	Giardiasis 14	0	0.113
174	Sparganosis 1	0	0.126
175	Sparganosis 2	0	0.166
176	Sparganosis 3	0	0.048
177	Sparganosis 4	0	0.120
178	Sparganosis 5	0	0.153
179	Paragonimiasis heterotremus 1	0	0.269
180	Paragonimiasis heterotremus 2	0	0.174
181	Paragonimiasis heterotremus 3	0	0.165
182	Paragonimiasis heterotremus 4	0	0.196
183	Paragonimiasis heterotremus 5	0	0.296
184	Paragonimiasis heterotremus 6	0	0.116
185	Paragonimiasis heterotremus 7	0	0.101
186	Paragonimiasis heterotremus 8	0	0.261
187	Paragonimiasis heterotremus 9	0	0.118
188	Paragonimiasis heterotremus 10	0	0.214
189	Taeniasis saginata 1	0	0.248
190	Taeniasis saginata 2	0	0.107
191	Taeniasis saginata 3	0	0.125

192	Taeniasis saginata 4	0	0.084
193	Taeniasis saginata 5	0	0.175
194	Taeniasis saginata 6	0	0.056
195	Taeniasis saginata 7	0	0.257
196	Taeniasis saginata 8	0	0.127
197	Taeniasis saginata 9	0	0.237
198	Hepatitis virus infection 1	0	0.293
199	Hepatitis virus infection 2	0	0.092
200	Hepatitis virus infection 3	0	0.169
201	Hepatitis virus infection 4	0	0.079
202	Hepatitis virus infection 5	0	0.237
203	Hepatitis virus infection 6	0	0.122
204	Hepatitis virus infection 7	0.5	0.148
205	Hepatitis virus infection 8	0.5	0.167
206	Hepatitis virus infection 9	0	0.177
207	Hepatitis virus infection 10	0	0.278
208	Hepatitis virus infection 11	0	0.271
209	Hepatitis virus infection 12	0	0.081
210	Hepatitis virus infection 13	0	0.080
211	Hepatitis virus infection 14	0	0.087
212	Hepatitis virus infection 15	0	0.075
213	Hepatitis virus infection 16	0	0.093
214	Negative control (Healthy person) 1	0	0.059
215	Negative control (Healthy person) 2	0	0.120
216	Negative control (Healthy person) 3	0	0.048
217	Negative control (Healthy person) 4	0	0.170
218	Negative control (Healthy person) 5	0	0.152
219	Negative control (Healthy person) 6	0	0.058
220	Negative control (Healthy person) 7	0	0.272
221	Negative control (Healthy person) 8	0	0.141
222	Negative control (Healthy person) 9	0	0.063
223	Negative control (Healthy person) 10	0	0.101
224	Negative control (Healthy person) 11	0	0.152
225	Negative control (Healthy person) 12	0	0.167
226	Negative control (Healthy person) 13	0	0.076
227	Negative control (Healthy person) 14	0	0.162
228	Negative control (Healthy person) 15	0	0.275
229	Negative control (Healthy person) 16	0	0.158
230	Negative control (Healthy person) 17	0	0.137
231	Negative control (Healthy person) 18	0	0.093
232	Negative control (Healthy person) 19	0	0.073
233	Negative control (Healthy person) 20	0	0.109
234	Negative control (Healthy person) 21	0	0.169
235	Negative control (Healthy person) 22	0	0.193
236	Negative control (Healthy person) 23	0	0.182
237	Negative control (Healthy person) 24	0	0.135
238	Negative control (Healthy person) 25	0	0.196
239	Negative control (Healthy person) 26	0	0.098
240	Negative control (Healthy person) 27	0	0.128

241	Negative control (Healthy person) 28	0	0.077
242	Negative control (Healthy person) 29	0	0.271
243	Negative control (Healthy person) 30	0	0.242
244	Negative control (Healthy person) 31	0	0.192
245	Negative control (Healthy person) 32	0	0.185
246	Negative control (Healthy person) 33	0	0.081
247	Negative control (Healthy person) 34	0	0.078
248	Negative control (Healthy person) 35	0	0.122
249	Negative control (Healthy person) 36	0	0.211
250	Negative control (Healthy person) 37	0	0.220
251	Negative control (Healthy person) 38	0	0.069
252	Negative control (Healthy person) 39	0	0.099
253	Negative control (Healthy person) 40	0	0.195
